# Genome wide identification, structural characterization and phylogenetic analysis of High-Affinity potassium (*HAK*) ion transporters in common bean (*Phaseolus vulgaris* L.)

**DOI:** 10.1186/s12863-023-01163-0

**Published:** 2023-11-14

**Authors:** Afrasyab Khan, Zamarud Shah, Sajid Ali, Nisar Ahmad, Maaz Iqbal, Arif Ullah, Firdous Ayub

**Affiliations:** 1 Department of Biotechnology, University of Science and Technology, Bannu, 28100 Pakistan; 2 Department of Biotechnology, Abdul Wali Khan University Mardan, Mardan, 23200 Pakistan; 3 Department of Biotechnology and Genetic Engineering, Hazara University, Mansehra, 21300 Pakistan; 4 Institute of Biotechnology and Genetic Engineering, University of Agriculture, Peshawar, 25130 Pakistan; 5 Department of Computer Science, Women University Swabi, Swabi, 23430 Pakistan

**Keywords:** Common bean, *HAKs*, Drought stress, Genome wide analysis, Expression analysis, Evolutionary analysis

## Abstract

**Background:**

High-Affinity Potassium ions represent one of the most important and large group of potassium transporters. Although *HAK* genes have been studied in a variety of plant species, yet, remain unexplored in common bean.

**Results:**

In the current study, 20 *HAK* genes were identified in common bean genome. Super-family “K_trans” domain was found in all *PvHAK* genes. Signals for localization of *PvHAK* proteins were detected in cell membrane. Fifty three *HAKs* genes, across diverse plant species, were divided into 5 groups based on sequential homology. Twelve pairs of orthologs genes were found in various plant species. *PvHAKs* genes were distributed unequally on 7 chromosomes with maximum number (7) mapped on chromosome 2 while only 1 *PvHAK* found on each chromosome 1, 4, and 6. Tandem gene duplication was witnessed in 2 paralog pairs while 1 pair exhibited segmental gene duplication. Five groups were made in *PvHAK* gene family based on Phylogeny. Maximum *PvHAKs* (10) were detected in Group-V while group-II composed of only 1 *PvHAK* gene. Variation was witnessed in number and size of motifs, and structure of *PvHAKs* associated with different groups. Light and hormone responsive elements contributed 57 and 24% share, respectively, to *cis* regulatory elements. qRT-PCR based results revealed significant increase in expression of all 4 PvHAK genes under low-potassium stress.

**Conclusion:**

The current study provides valuable information for further functional characterization and uncovering the molecular mechanism associated with Potassium transportation in plants.

**Supplementary Information:**

The online version contains supplementary material available at 10.1186/s12863-023-01163-0.

## Introduction

Potassium is one of the most important macronutrients which accounts for up to 10% of plants dry weight [1]. Decrease in amount of potassium below 10 g/kg of dry weight is associated with severe defects in plant growth and development [2]. Metabolism of protein, carbohydrate and activities of enzymes are some of the biochemical processes affected by potassium. Similarly physiological processes including regulation of stomata and photosynthetic operation are halted by inadequate supply of potassium [3]. Moreover, potassium acts as main player in enhancing plant resistance to a number of biotic and abiotic stresses [4]. More than 50% decline in soybean yield has been witnessed, due to potassium deficiency, across a wide range of soil type. **(Louisiana State University soil fertility specialist Rasel Parvej**). Stunted growth that eventually leads to reduced productivity is one the major symptom associated under supply of potassium.

A large number of K^+^ channels and transporters have been designated to carry out the absorption and translocation of potassium inside the plants [5]. High potassium concentration has been recorded as the driving force for K^+^ channels to undertake its transportation with comparatively low efficiency. On the other hand, K^+^ transporters are good enough to draw and transport the nutrients at low external concentrations and were truly called as high affinity system [6]. In plants, potassium transporters have been grouped into 4 families: KT (K^+^ transporter)/HAK (high-affinity K^+^), Trk (Transport of K^+^), KEA (K^+^ efflux anti-porter), and CHX (cation/hydrogen exchanger) [7]. The size of HAK proteins ranged from 300 to 900 amino acid have pronounced role in K^+^ transport. Among the legume crops, common bean *(Phaseolus vulgaris*) is regarded as the most important crop grown for human consumption at global level [8]. Historically a large number of developing countries across Africa, Latin America, and Asia have remained as the main consumer of common bean [9]. Even today, common bean is rated within the top ten most consumed vegetables/pulses worldwide [10]. Common bean is nutritionally rich with high concentration of protein, fiber, iron, magnesium and folate [11].

Potassium (K) is one of the major yield limiting nutrients of common bean in South America [12]. The growth of common bean varieties with efficient K transportation system supplemented with K fertilizer would prove helpful in improving the yield and reduce the production cost. Since, common bean have shown better response to the uptake and transportation of potassium, thus, this crop is assumed to have repository gene for K^+^ transportation. Though, some genomic data of common bean is available in literature, yet, little is known about of *HAK* transporter family in common bean. The objective of current work was to identify *HAK* transporter genes across the genome of common bean and characterize for important physiochemical, structural, phylogenetic and expression analysis.

## Materials and methods

### Detection of *HAK* gene family in *Phaseolus vulgaris* genome

*HAK* sequence of *Arabidopsis thaliana* (NP_187864.1) was taken from “National Centre Biotechnology Information”. The presence of *HAK* domain was found in the sequence of *Arabidopsis thaliana* using Pfam finder (http://pfam.sanger.ac.uk [13]. Domain of *AtHAK* was utilized for obtaining *HAK* transcripts from genome of common bean using online phytozome v.13 database (https://phytozome-next.jgi.doe.gov) [14]. Short and redundant protein sequences were removed. Motif finder (online tool) was used for confirmation of *HAK* domain in the transcripts.

### Physiochemical characterization of *PvHAK* genes

Online database phytozome (https://phytozome-next.jgi.doe.gov.) [14] was used for finding start-end point, strand nature (forward or reverse), CDS and protein length. Likewise, various other physical characters including molecular weight, PI, GRAVY, Instability index and aliphatic index of protein sequences were uncovered by subjecting the data to expasy protparam (online tool: https://web.expasy.org/protparam) [15].

### Conserved domains within the protein sequence of common bean (domain architecture)

Rename file and Hitdata files were generated by using TBtool.v1.09854 software [16] and conserved domain database [17] respectively. Domain architecture [16] was generated by subjecting these files to TBtool.v1.09854.

### Predicted subcellular localization of *PvHAKs* protein

Protein sequences of all *PvHAKs* were subjected to online tools CELLO Life and Wolf Psort for predicting their subcellular localization. Heatmap was generated using TBtool [16] for visualization of protein in the cell using Wolf Psort method.

### Phylogenetic, motif and Gene Structure analysis of *PvHAK* family

Molecular Evolutionary Genetics Analysis (MEGA 7) software [18] was used for multiple alignments of protein sequences. Maximum Likelihood (ML) approach with 1,000 bootstrap replicates was adopted for exploring phylogenetic relationship among members of *PvHAK* gene family. Conserved motifs in protein sequences of *PvHAK* family were visualized by using online tool MEME (http://memesuite.org) [19]. Maximum number of 10 motifs per sequence was adjusted by default and subjected to Pfam database (http://pfam.sanger.ac.uk) [13] for annotation analysis. Online database pytozome was used for extracting genomic and CDS sequence of *PvHAK* genes. Structural features of *PvHAK* genes were shown using Gene Structure Display Server 2.0 (GSDS).

### Comparison of HAK genes among different species

Comparative analysis of *HAK* genes across diverse plant species including *P. vulgaris, A. thaliana, Z. Mays, O. sativa, H. vulgare, S. lycopersicum, C. annum, P. australis, P. patens, C. nodosa and T. halophila*, was carried out using MEGA7.0.26 software [18]. Two genes from the same species placed in the same clade were considered as paralog while those belong to different plant species were designated as orthlogs. Both paralog and ortholog gene pairs have high level of homology.

### Chromosome mapping, duplication, Ka/Ks ratio and selection analysis of *PvHAKs*

The information necessary for chromosome mapping such as chromosome number, position and length of *PvHAK* genes on chromosome was obtained from phytozome [14] (online database) The data was incorporated in PhenoGram Plot (http://visualization.ritchielab.psu.edu/phenograms/plot) for mapping genes on the respective chromosomes [20]. Online tool SIAS (http://imed.med.ucm.es › Tools › sias) was used to determine the level of identity (homology) between 2 genes. The coding genes with ≥ 50% identity and covering ≥ 90% protein length were regarded as duplicated genes [21]. Further analysis of genes with same species and clade of phylogenetic tree was carried out for determining the pattern of duplication (tandem or segmental). All the homologous genes with physical distance of < 50 Kilobase (kb) were denoted as tandemly duplicated genes while 2 genes separated by more than 50 Kilobase (kb) were considered as segmentally duplicated genes [22]. The values of non-synonymous (Ka) and synonymous (Ks) substitutions, of all segmentally duplicated gene pairs, were obtained from online available Plant genome duplication database [23]. The Ka/Ks ratio was analyzed for determining selection pressure on duplicated genes. Less than 1 Ka/Ks ratio was regarded as less negative selection while greater than 1 value were considered as positive selection. The values equal to 1 added to neutral evolution. TBTool [16] was used for getting Ka and Ks values of the PVHAKs paralogous pairs. Selecton version 2.2 was utilized to find amino acids within the PVHAKs proteins under positive and purifying selection pressure [24].

### Promoter region analysis

The 1500 bp upstream sequence from the translation initiation site (ATG) of each *PvHAK* gene was obtained from Phytozome and analyzed for *cis*-regulatory elements using online tool Plant Cis-Acting Regulatory Element (PlantCARE); [25].

### Growing plant materials, low-potassium stress treatments and qRT-PCR

Transcriptomic/expression data of common bean was extracted from Gregorio Jorge et al., 2020, GSE123381 [26] using NCBI GEO database. The data was filtered for transcripts belong to HAK family using TBtool software. Commom bean (cv: Gorilla) seeds were sterilized with sodium hypochlorite (10%) for 15 min and rinsed with tap water for 30 min [27]. The seeds were germinated on basic salt medium (BSM, 0.5mM KCl + 0.1 mM CaCl2) for 2 d, and then BSM was changed to Hoagland solution (20%) for another 8 d in growth room with a photoperiod of 14/10 h, light intensity of 200 ± 25 µmol·m^− 2·s− 1^, temperature of 23/18°C (day/night) and relative humidity of 60%. Ten days old seedlings were subjected potassium stress (0.01 mMK+) [28] in background of Hoagland solution (20%) while seedlings growing in Hoagland solution (20%) were marked as control. The solutions were renewed every 2 days. After treatments for 1 h, 3 h, 6 and 9 h samples from both treated and control plants were collected for RNA extraction and qRT-PCR. All samples were taken in three replicates.

Total RNA was extracted from the samples using MiniBEST Plant RNA Extraction Kit (9769, TaKaRa, Japan) following the manufacturer’s instructions. The cDNA was synthesized from total RNA (1 µg) using PrimeScript RT Master Mix (RR036A, TaKaRa, Japan) and was used as templates for qRT-PCR amplification. qRT-PCR amplification was performed with LightCycler 480 II (Roche, Basel, Switzerland) using iTaq Universal SYBR Green Supermix (1,725,124, Bio-Rad, USA). The relative gene expression was calculated based on the 2^−△△CT^ method using actin as the internal standard [29]. Primers used for qRT-PCR are listed in Supplementary Table S7.)

### Statistical analysis

The experiments were performed in triplicate and the data was subjected to student’s t-test for analysis. The data with the error bars represent 95% confidence interval.

## Results

### **Genome wide identification of*HAK*** **gene family members in common bean and their physico-chemical characterization**

**Fig. 1:**
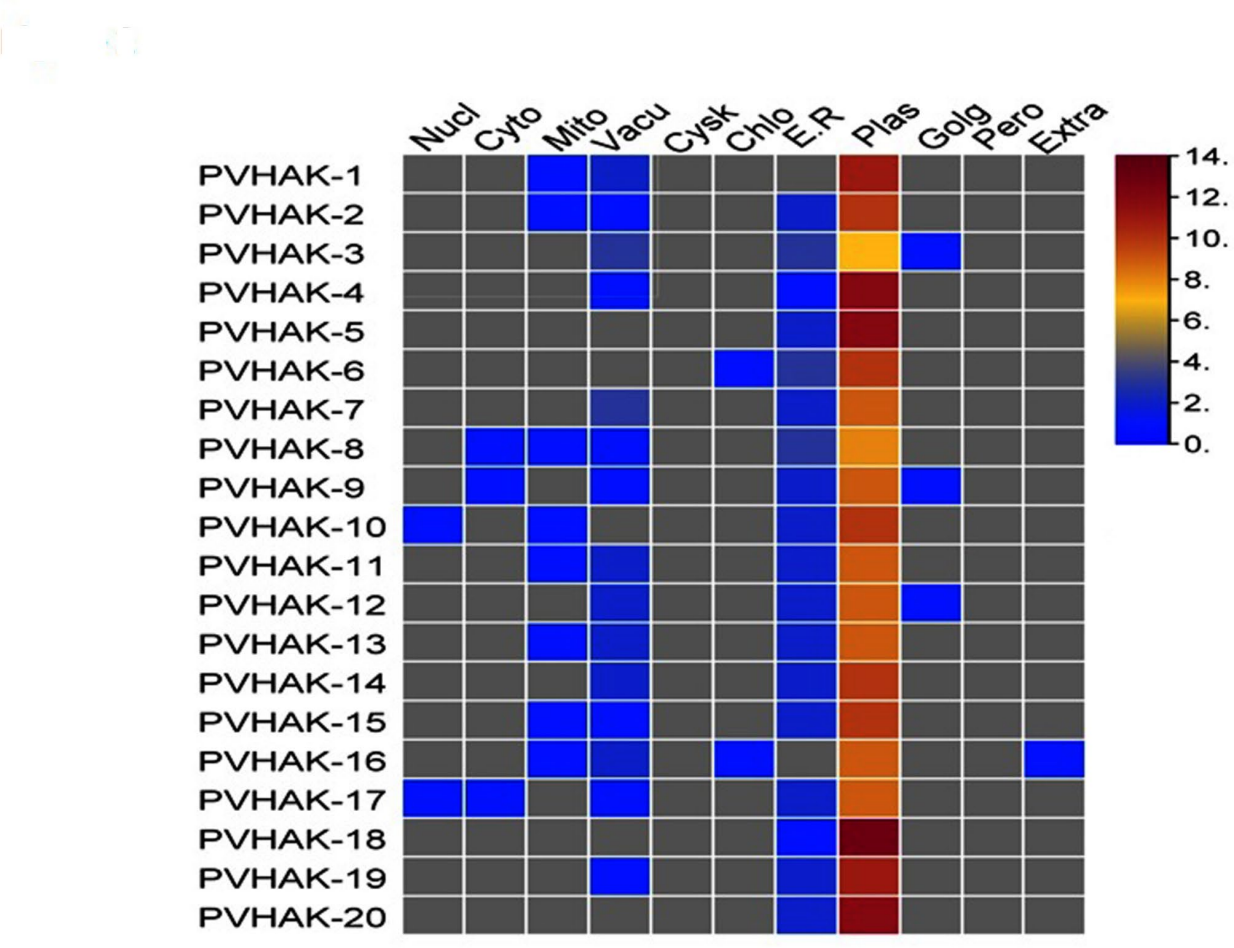
Wolf PSORT based sub-cellular localization of *PvHAK* proteins. Quantitative heat map of *PvHAK* gene represents sub-cellular localization. Red color bar represents high number of proteins, blue represents low numbers and yellow shows intermediate number of proteins

A total of 20 *HAK* unique full length genes were identified in common bean genome and were designated as *PvHAK-1* to *PvHAK-20* on the basis of location on chromosome in ascending order (Table S1). The CDS sequence, protein length and molecular weight of *PvHAK* genes ranged from 1047 to 2541 (bp), 348–846 (aa) and 39.6–94.1 (Kda), respectively (Table S1). Start-end point and strand (forward or reverse) of each transcript have been presented in the Table S1. Maximum isoelectric point (PI, 9.44) and GRAVY (0.44) were witnessed in *PVHAK-6* and *PVHAK-14*, respectively. Similarly, 6 *PvHAK* proteins exhibited higher than 40 instability index while *PvHAK-12* was recorded to have the highest aliphatic index (111.32). For subcellular localization, heat map generated through wolf psort (online tool) revealed a wide range (7–13) of *PVHAK* proteins in the plasma membrane. Maximum signals (13) were detected for *PvHAK-18* while *PvHAK-3* emitted minimum signals (7) in plasma membrane (Table S2, Fig. 1). Cello life (online tool) based analysis predicted the localization of all *PvHAK* proteins in the plasma membrane (Table S1). Trans-membrane (TM) helices in each *PvHAK* protein were found out. Maximum TMs (14) were found in 2 genes (*PvHAK-4* and *PvHAK-19*) while only 3 TMs were detected in PvHAK-16. The number of TM helices in other PvHAK genes fall in the range of 6–13 (Table S1).

### Phylogenetic analysis of *HAK* genes across the genomes of different plant species

Phylogenetic analysis of HAK gene family was carried out across 11 plant species including *P. vulgaris, A. thaliana, Z. Mays, O. sativa, H. vulgare, S. lycopersicum, C. annum, P. australis, P. patens, C. nodosa, and, T. halophila*. A total of 53 *HAKs* were shown in phylogenetic tree with 5 subgroups (I, II, III, IV and V). Subgroup-V was found to be largest one with 16 genes, followed by subgroup-I and subgroup-IV with 13 and 10 genes respectively. Each of subgroup-II and subgroup-III contains the least number of 7 genes. Three paralog gene pairs (*PvHAK-6–PvHAK-7, PvHAK-16–PvHAK-18, and, PvHAK-5–PvHAK-12*) were witnessed in *PvHAK* family. Similarly, 12 ortholog gene pairs were identified in different plant species. Maximum numbers of 7 orthologs pairs were found in *P. vulgaris and A. thaliana*. One pair each was found in *C. nodosa* -*O. sativa*, *P. australis*- *Z. mays, S. lycopersicum- C. annum, O. sativa-H. vulgare and A. thaliana-T. halophila.*

**Fig. 2:**
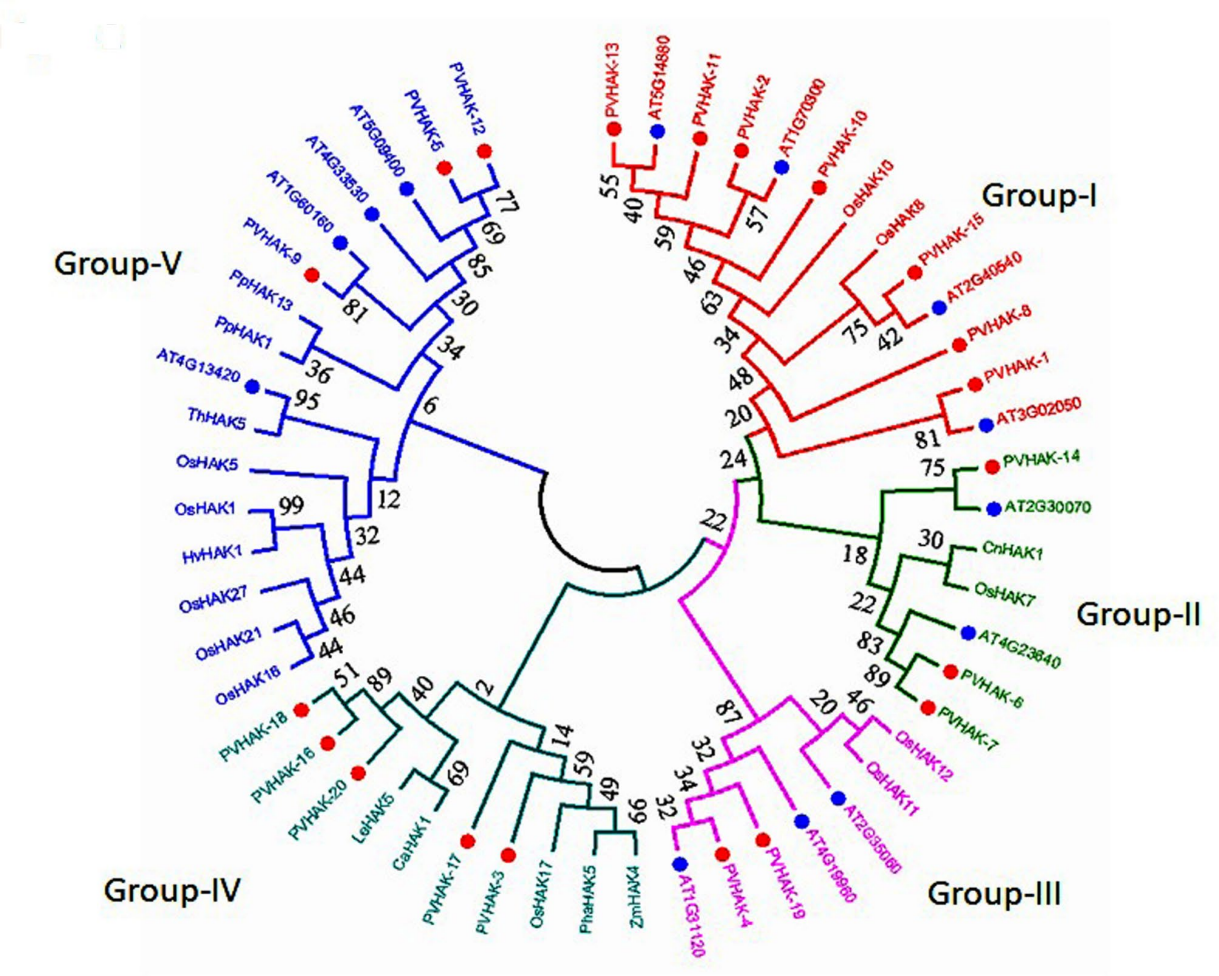
Comparative phylogenetic analysis of *HAK* proteins across different plant species. Bootstrap values in percentage are indicated on the nodes. Different groups are highlighted with different colors

*Z. mays, O. sativa, H. vulgare, S. lycopersicum, C. annum, P. australis, P. patens, C. nodosa and T. halophila* were recorded to have no common ancestral gene (ortholog pair) with host plant (*P. vulgaris*) and thus were regarded as out groups in the present study (Fig. 2).

### Gene location on chromosome, duplication analysis, Ka/Ks ratio and positive selection analysis

**Fig. 3:**
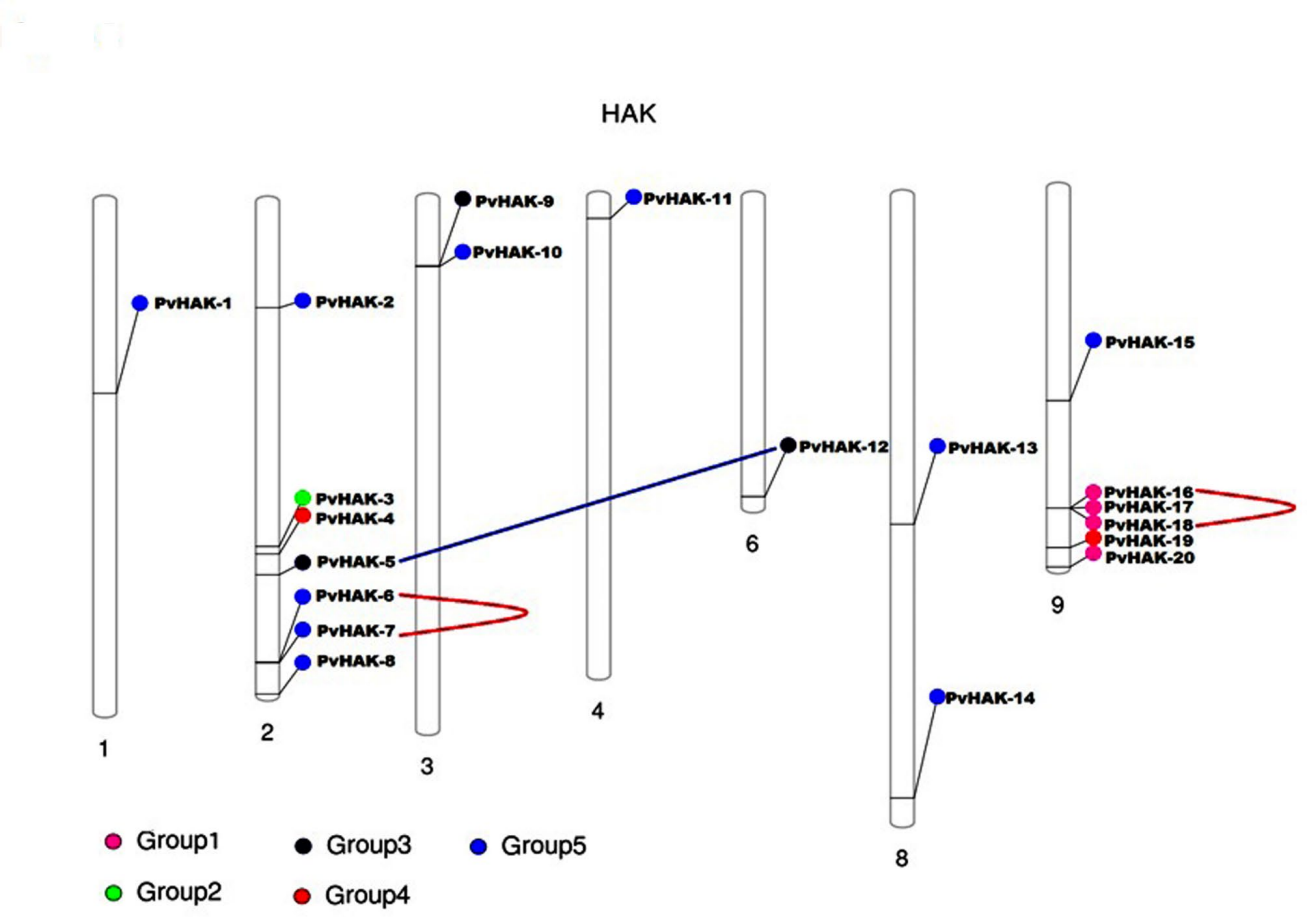
Chromosomal locations and duplications pattern of VUHSP70 genes across cowpea genome. Chromosome number is mention below each bar while its size is indicated by relative length taken from phytozome. The circles marked with different colors on each chromosome represent the genes belong to specific group. Paralog pairs with tandem gene duplication are joined by pink curved pink lines while those with segmental duplication is connected by blue straight line

**Fig. 4:**
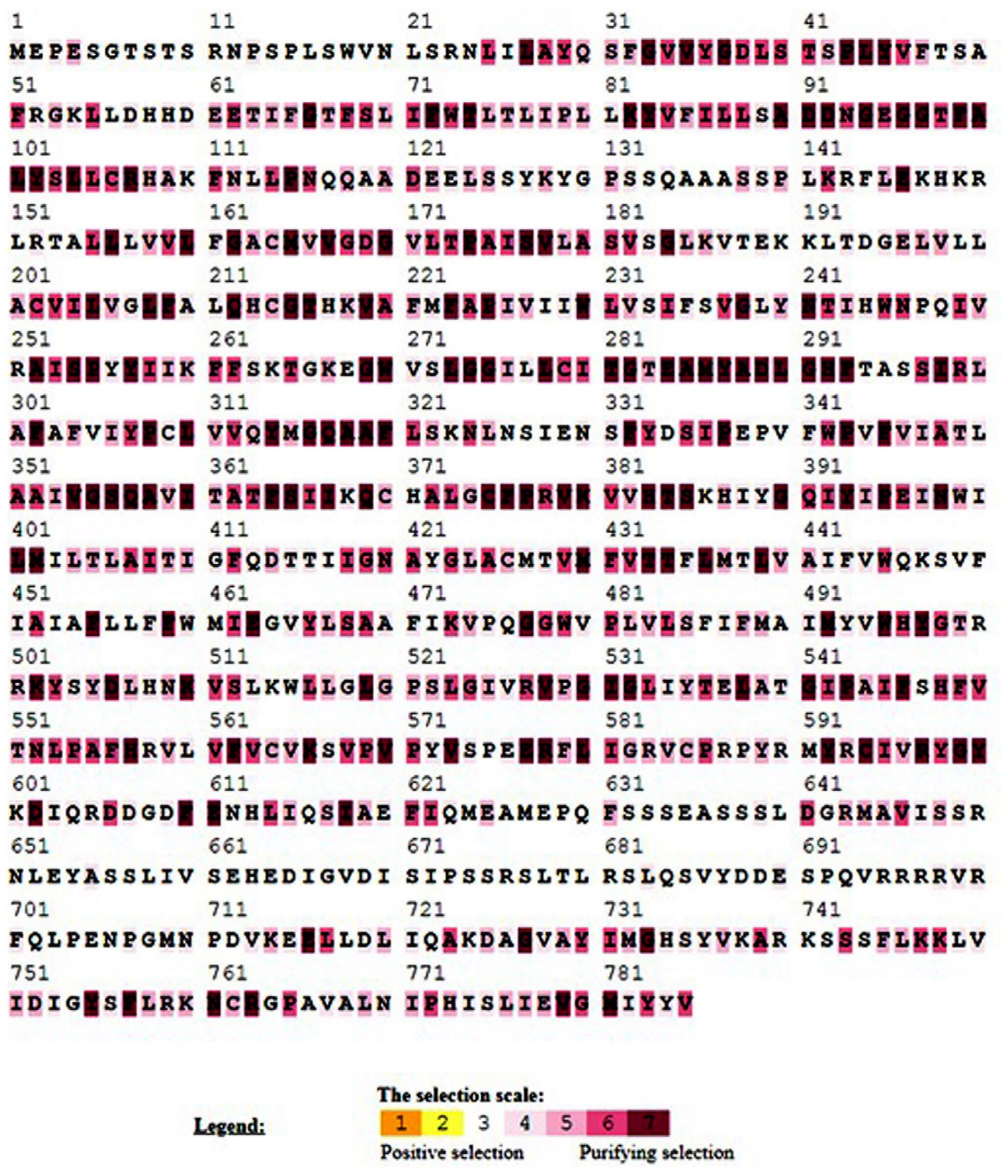
Analysis of evolutionary selection pressure on amino acids of *PvHAK* protein. Yellow and brown highlight positive selection, gray and white represent neutral selection while purple indicates purifying or negative selection pressure

Twenty genes identified across the common bean genome were mapped unequally on 7 chromosomes. Maximum number of genes (7) were found on chromosome 2 while chromosomes 1, 4, and 6 revealed minimum number of genes. Similarly, results showed the presence of 6 genes on chromosome 9 and 2 genes on each of the chromosomes 3 and 8 (Fig. 3). Two paralog pairs (*PvHAK-6–PvHAK-7 and PvHAK-16–PvHAK-18*) exhibited a physical distance of 30.459 and 13.974 Kb, respectively, and, hence were marked as tandemly duplicated genes. On the other hand, one paralog gene pair (*PvHAK-5–PvHAK-12*) revealed a physical distance of 7403.474 Kb and was counted as segmentally duplicated gene pair (Fig. 3). The Ka/Ks ratio of duplicated genes ranged from 0.10 to 0.30 (less than 1 (Table S3) suggesting the passage of paralogous *PvHAK* pairs under purifying process. Consistently evolving location of amino acid is very important for conservation of protein structure and function. In the current study, 471 out of 785 amino acids of *HAK* protein were under purifying selection while the rest (314) were influenced with neutral selection (Fig. 4).

### Phylogenetic analysis, Exon/intron, motifs and domain structure

**Fig. 5:**
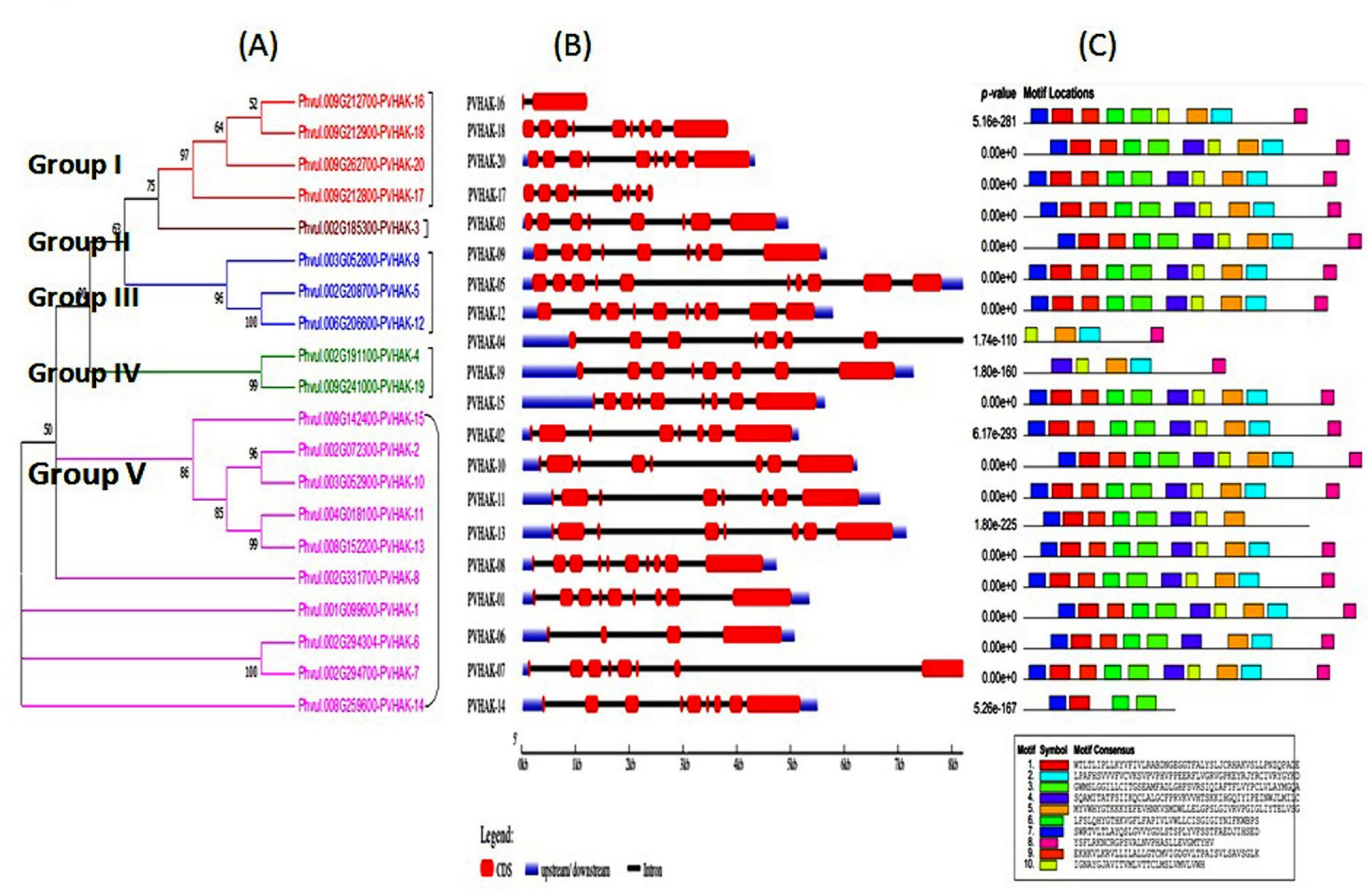
Phylogenetic, structural and conserved motif analysis of *PvHAK* genes **A)** Group of Phylogenetic tree highlighted with different colors (5 groups) **B)** Red boxes represent exons/CDS (coding DNA sequence), black lines show introns while un-translated region (UTR) highlighted with blue boxes **C)** Conserved motifs of PvHAK proteins are shown as colored boxes. The scale used is given at the bottom [kilobase (kb)]

**Fig. 6:**
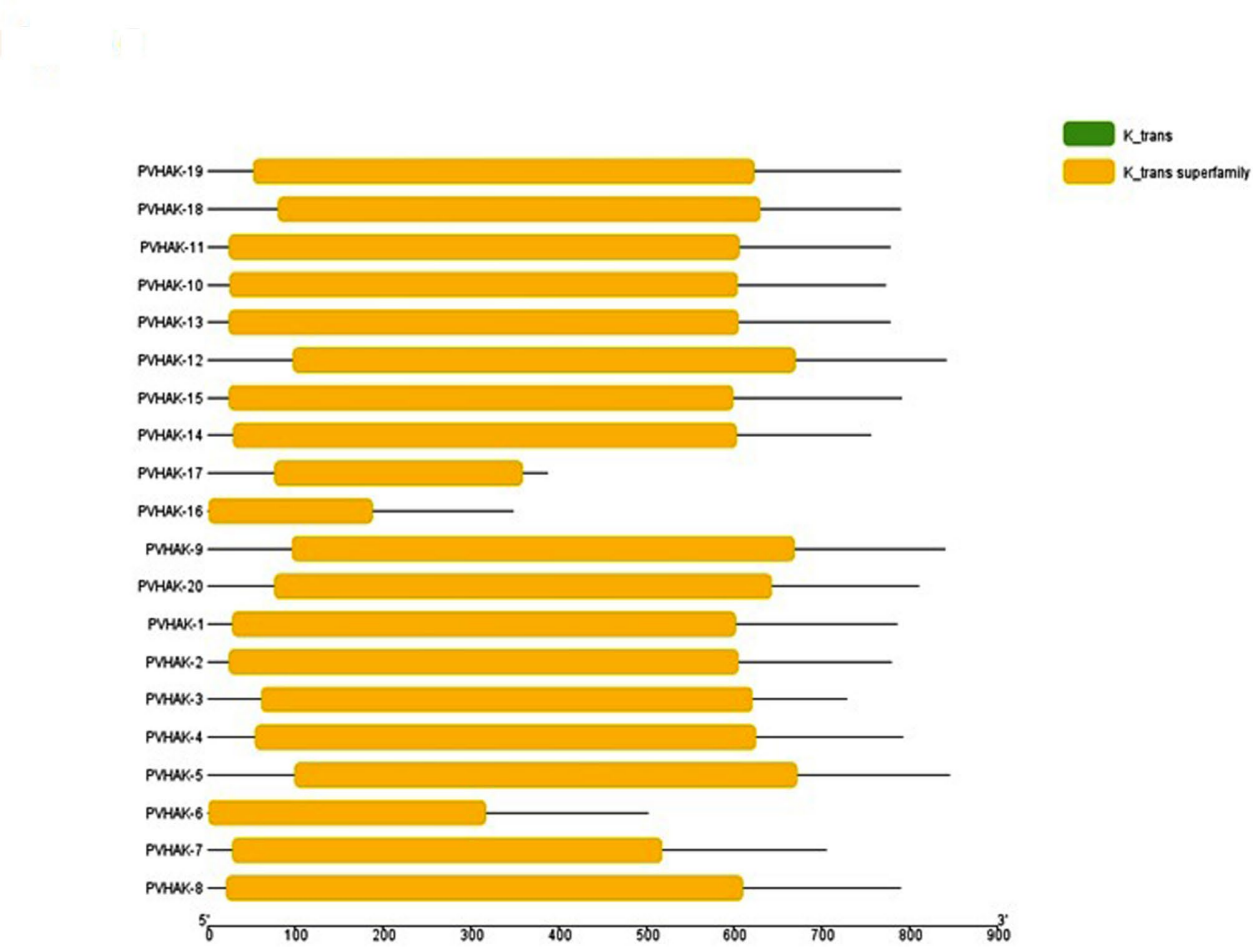
Domain analysis of *PvHAK* proteins. The rectangular yellow boxes represent K_trans superfamily domains

Maximum likelihood based Phylogenetic tree of 20 *PvHAK* genes was constructed for showing evolutionary divergence. *PvHAK* gene family was grouped into 5 clusters using phylogenetic tree. Subgroup-V was found to be the largest one with 10 *PvHAK* genes followed by subgroup-I, III and IV with 4 and 3 and 2 *PvHAK* genes, respectively. *PvHAK-3* was recorded as the sole gene belongs to subgroup-II (Fig. 5A). Variation was witnessed in structure of genes belong to different subgroups. In subgroup-I, each of *PvHAK-18* and *PvHAK-20* were found to contain 9 exons and 8 introns while *PvHAK-17* revealed 8 exons and 7 introns. *PvHAK-16* behaves differently in the group with only 2 exons and 1 intron. The sole genes belong to subgroup-I (*PvHAK-3*) showed 8 exons and 7 non coding intervening sequences. In subgroup-III, each of *PvHAK-05* and *PvHAK-12* were found to contain 10 exons and 9 introns while *PvHAK-9* was recorded with 9 exons and 8 introns. Each gene belong to subgroup-IV (*PvHAK-04* and *PvHAK-19*) were detected with 7 introns. A wide range of exons (4–10) and introns (3–9) was witnessed in subgroup-V. Highest number of exons (10) and introns (9) were detected in *PvKAH-10* while *PvHAK-06* shown the minimum exons (4) and introns (3) (Fig. 5B). In subgroup-I, each of *PvHAK-18*, *PvHAK-20* and *PvHAK-17* revealed 10 motifs while 9 motifs were detected *PvHAK-16*. Similarly, *PvHAK-3* of subgroup-II showed 10 motifs. Two genes of subgroup-III (*PvHAK-9* and *PvHAK-5*) exhibited 10 motifs while *PvHAK-12* showed lowest number of motif (4) in the subgroup. *PvHAK-19* of group IV showed 10 motifs while 5 motifs were detected in *PvHAK-4*. A wide range of motif (4–10) was witnessed in subgroup V. Seven out of 10 genes shown 10 motifs while *PvHAK-14* showed the lowest number of 4 motifs (Fig. 5C). The size of 20 *PvHAK* predicted motifs ranged from 29 to 50 amino acids. Five out of 10 motifs (1, 2, 3, 4 and 5) showed maximum length with 50 amino acids followed by motif 6, 7 and 9 with 41amino acids. Motif 8 was found to contain 31 amino acids. Minimum length of 29 amino acids was exhibited by motif 10 (Table S4). All *PVHAK* proteins have only potassium transporter domain (Fig. 6).

### Promoter analysis

**Fig. 7:**
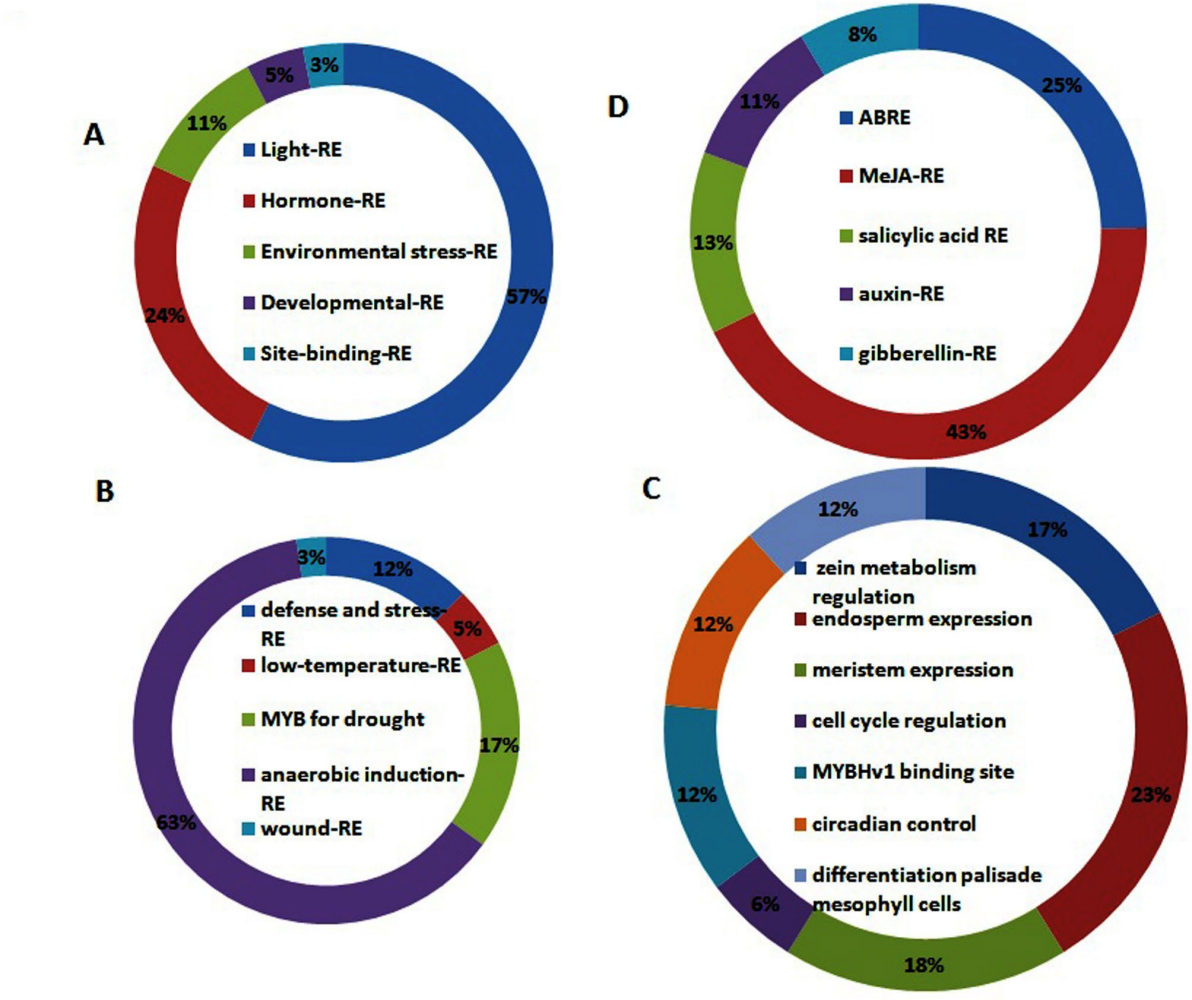
Promoter region (1500 bp upstream) analysis of *PvHAK* genes. **(A)** Percentage of different cis-elements **(B)** Percentage of different stress responsive cis-element **(C)** Percentage of different hormone responsive cis-element **(D)** Percentage of different development related cis-element

Promoter region of *PvHAK* genes raveled a total 380 cis regulatory. Light and hormone responsive elements occupied 57% (218 elements) and 24% (93 elements) of cis regulatory elements, respectively. Environmental stress, development and site-binding related elements made 10% (40), 4% (17) and 3% (12), respectively, of cis elements in upstream region of *PvHAK* (Table S5, Fig. 7A). Among environmental stress related elements, 63% were composed of anaerobic induction regulatory elements (AIRE) followed by drought related (MYB) with 17% and defense-stress related element with 12% elements. Low temperature and wound related elements constituted only 5 and 3% elements, respectively (Fig. 7B). Among development related cis acting element, highest percentage of endosperm related element (23%) was detected followed by meristem related (18%) and zein metabolism related elements (17%). Each of the 3 kind of elements i.e. MYBHv1 binding site, palisade mesophyll cells differentiation and circadian control, were found to constitute 12% elements. Only 6% elements involved in cell cycle regulation were detected (Fig. 7C). Among the hormone related elements, 43% were detected as MeJA-RE, followed by ABRE with 25% and salicylic related elements with 13% elements. Axuin and gibbereline-RE were detected to constitute 13 and 11% elements, respectively (Fig. 7D).

### Expression analysis of PvHAK genes under low-potassium stress

**Fig. 8:**
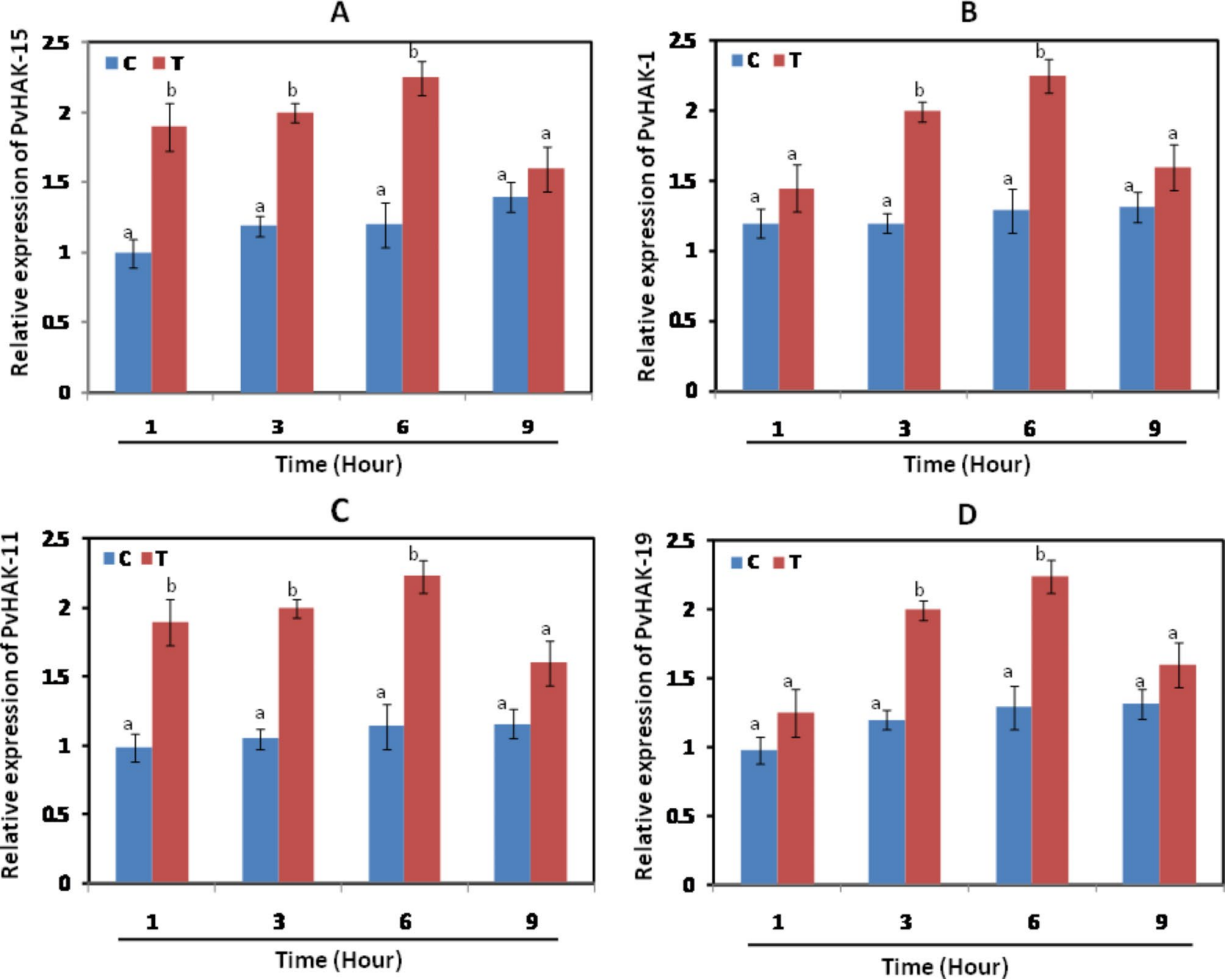
Expression profiling of four PvHAK genes in response to potassium deficiency (0.01mMK+). Lowercase letters indicated the significant difference at p < 0.05

According to transcriptomic data of common bean extracted from Gregorio Jorge et al. [26], PvHAK-15 showed maximum expression in 5 major tissues including flower buds, leaves, stem, young pods and young trifoliate. Similarly, an elevated level PvHAK-11 expression was recorded in young trifoliate, young pods, leaves and flowers buds with maximum expression detected in flowers. Likewise, PvHAP-19 and PvHAK-1 exhibited higher expression in all the tissues investigated under low-potassium stress. Some other genes including PvHAK-9, PvHAK-12, PvHAK-13 and PvHAK-14 presented high expression upon exposure to low potassium stress (Table S6). The expression profile of 4 genes including PvHAK-1, PvHAK-11, PvHAK-15, PvHAK-19 under potassium stress was investigated to validate the results experimentally. Significant elevation in expression of PvHAK-15 and PvHAK-11 after 1, 3 and 6 h K treatment, compared to control, was witnessed. However, no such increase was observed after 9 h K treatment (Fig. 8A, C). Similarly, relative expression of PvHAK-1 and PvHAK-19 was significantly enhanced, compared to control, after 3 and 6 h treatment however, non significant increase in their expression was recorded after 1 and 9 h treatment (Fig. 8B, D).

## Discussion

Potassium is one of the most indispensible micro-nutrients required for plant growth and development. An efficient transportation system within the plant is as important as availability of potassium in rhyzosphere. *HAK* has been reported as the largest gene family responsible for K + transportation [30].

The *HAK* family was first detected in bacteria [31] followed by kingdom Plantae and Fungi [32] but were not reported in Protista and Animalia [33]. In plantae, both monocots and dicots were found to have homologues of *HAK* in the form of multigene families [34]. In the current study, a comprehensive set of 20 unique full length *HAK* genes was identified in common bean’s genome which is more than those reported in barley (5) and Arabidopsis (13). The strength of *HAK* genes family in common/french bean was found less than poplar (21) [35], rice (27) [9] and maize (27) [36]. The uneven mapping of 20 *PvHAK* genes on 7 out of …. chromosomes indicate that gene duplication has occurred in common bean genome. These results are in agreement with [37]. Gene duplication as evident in the present study has been reported to be the main cause for expansion gene families of plants [38]. The presence of “K_trans” super-family domain in all *PvHAKs* further strengthens the role of this family in K- transportation. The localization of *PvHAK* proteins in the plasma membrane, as found in the current study, provides strong channels for passage of these K ions across the membrane.

Twenty members of *PvHAK* gene family were clustered into 7 groups based phylogenetic analysis. A narrow range of exon/introns was recorded in *HAK* gene family. The number of exons in common bean *PvHAK* genes ranged from 2 to 10 which is very similar to that of rice (2–10) [9] and close to maize (3–10) [36]. The *PvHAK* genes on account of its length were detected to decode proteins with 348 to 846 amino acids. Of these, 569 amino acids constitute the characteristic “K_trans” domain (PF02705, in Arabidopsis T). Varied number and size of motifs in *PvHAKs* were detected with 4–10 and 29–50 amino acids, respectively. Nine out of 10 motifs were detected to have role in potassium transportation except motif 8 with unknown function. The motifs with different features may contribute to divergence of *PvHAKs*. Results revealed that members belong to the same groups shares the same gene features and motif configuration. Contrary to this, variation was observed in number of coding and non coding regions of *PvHAK* genes associated with different groups. This difference in expression pattern of *PvHAK* genes strengthened the view that gain or loss of introns during evolution might be the causal agent of such variability.

During comparative analysis in diverse plant species, 53 *HAKs* were divided into 5 groups based on maximum likelihood. Twelve pairs of orthologous genes detected in different plants exhibited that these genes have been derived from a common ancestral gene. Arabidopsis thaliana was found as the closest relative to P. vulgaris on account of sharing maximum numbers of orthologous pairs (7). The other crops (*Z. Mays, O. sativa, H. vulgare, S. lycopersicum, C. annum, P. australis, P. patens, C. nodosa* and *T. halophila*) exhibited no ortholog pair with the host plant and thus were regarded out groups. The presence of one pair of orthlog as witnessed in each of the 5 combinations (*C. nodosa and O. sativa, P australis and Z. mays, S. lycopersicum and Capsicum annum, Oryza sativa and Hordeum vulgare, and, Arabidopsis thaliana* and *Thellungiella halophila*) suggest some degree of resemblance. Similarly, the detection of 3 paralogous exhibited gene duplication within *PvHAKs* gene family.

In the current study, 380 cis regulatory elements in upstream regions of 20 *PvHAK* genes were detected. Light and hormone responsive elements were found as the major contributor to cis regulatory elements with 57% (218 elements) and 24% (93 elements) share, respectively. Ten (40), 4% (17) and 3% (12) of cis regulatory elements occupied by environmental stress related, development related and Site-binding related elements, repectively. These results are in line with various stress and hormones responsive elements reported in promoter region of many HAKs [39]. A huge number of promoter related cis regulatory elements (1410) were found in the promoter region of *PvHAKs*. Among the cis acting light responsive elements, conserved DNA module (G-box), cis-acting regulatory elements (ACE), light responsive element (GT1-motif) and part of a light responsive element shared 97, 28, 22 and 58 elements, respectively. Abscisic acid responsive elements (ABRE), methyl jasmonate-responsive element (CGTCA motif & TGACG motif) and salicylic responsive elements (TCA-element) were found in 40, 23, and 12 number, respectively. Very few auxin, gibberline responsive elements and no ethylene responsive elements (ERE) were detected in the promoter region of *PvHAK* genes. Maximum number of hormone responsive elements was found in promoter region of *PvHAK-15*. Anaerobic inducing responsive element (ARE) is the major stress responsive cis acting elements detected in the current study. Time-dependent increase in expression of all 4 genes (PvHAK-1, PvHAK-11, PvHAK-15, PvHAK-19) after low-potassium treatment for 1, 3 and 6 h treatment was found in line with earlier reports in rice [40, 41]. These results strengthened earlier findings of Gregorio Jorge et al. (2020) [26]. A common pattern of decline in expression of all 4 genes after exposure to potassium stress for 9 h could possibly be attributed to negative feedback mechanism and cross-talk with other pathways. These results were found in agreement with previous report in barley [37]. Up-regulation of HAK genes, under potassium stress, enhances the uptake of potassium by the roots and translocation to other parts of the plants [42, 43]. The absorption of more potassium in response to elevated expression of HAK genes not only assist optimum plant growth but also help plants to encounter the detrimental effect of different stresses by invoking the ROS scavenging system. These results strengthened earlier reports in two different crops, tea [44] and willow [45].

## Conclusion

Twenty *HAK* genes in the common bean genome were mapped unequally on 7 chromosomes. Fifty three HAK detected across diverse plant species were divided into 5 groups. Twelve pair of orthologs and 3 pair of paralog were detected during comparative analysis. Tandem duplication was witnessed in 2 paralog pair while 1 paralog pair was segmentally duplicated. Five groups of *PvHAK* gene family were made based on phylogenetic analysis. Variation was observed in number and size of motifs and structure of *PvHAKs* associated with different groups. Light and hormone responsive elements were found as the major contributor to *cis* regulatory elements. Expression profile of *PvHAK* genes under low-potassium stresses revealed that many genes of this family are involved in uptake and translocation of potassium across the plant body.

### Electronic supplementary material

Below is the link to the electronic supplementary material.

Supplementary Material 1

Supplementary Material 2

Supplementary Material 3

Supplementary Material 4

Supplementary Material 5

Supplementary Material 6

Supplementary Material 7

## Data Availability

The data is available in the respective web links and accession numbers. *HAK* sequence of *Arabidopsis thaliana (*Accession No: NP_187864.1). National Centre Biotechnology Information (https://www.ncbi.nlm.nih.gov/). Pfam finder (http://pfam.sanger.ac.uk). Phytozome v.13 database (https://phytozome-next.jgi.doe.gov. ). Expasy protparam (online tool: https://web.expasy.org/protparam). (TBtool.v1.09854 software) https://github.com/CJ-Chen/TBtools/releases. MEME online tool (http://memesuite.org). Pfam database (http://pfam.sanger.ac.uk). PhenoGram Plot (http://visualization.ritchielab.psu.edu/phenograms/plot). Online tool SIAS (http://imed.med.ucm.es › Tools › sias).
